# SOMAscan-based proteomic measurements of plasma brain natriuretic peptide are decreased in mild cognitive impairment and in Alzheimer's dementia patients

**DOI:** 10.1371/journal.pone.0212261

**Published:** 2019-02-14

**Authors:** Edin Begic, Suncica Hadzidedic, Ajla Kulaglic, Belma Ramic-Brkic, Zijo Begic, Mirsada Causevic

**Affiliations:** 1 Department of Pharmacology, Sarajevo Medical School, Sarajevo School of Science and Technology, Sarajevo, Bosnia and Herzegovina; 2 Department of Cardiology, General Hospital "Prim.Dr. Abdulah Nakas", Sarajevo, Bosnia and Herzegovina; 3 Computer Science and Information Systems Department, Sarajevo School of Science and Technology, Sarajevo, Bosnia and Herzegovina; 4 Department of Pediatric Cardiology, University Clinical Center, Sarajevo, Bosnia and Herzegovina; Universitatsklinikum Leipzig, GERMANY

## Abstract

Alzheimer's disease represents the most common age-related neurodegenerative disorder and a leading cause of progressive cognitive impairment. Predicting cognitive decline is challenging but would be invaluable in an increasingly aging population which also experiences a rising cardiovascular risk. In order to examine whether plasma measurements of one of the established biomarkers of heart failure, brain natriuretic peptide (BNP), reflect a decline in cognitive function, associated with Alzheimer's disease neurodegeneration, BNP levels were analysed, by using a novel assay called a SOMAscan, in 1. cognitively healthy, control subjects; 2. subjects with mild cognitive impairment, and 3. subjects with Alzheimer's disease. The results of our study show that the levels of the BNP were significantly different between the three types of diagnoses (*p* < 0.05), whereby subjects with mild cognitive impairment had the lowest mean BNP value, and healthy subjects had the highest BNP value. Importantly, our results show that the levels of the BNP are influenced by the presence of at least one *APOE4* allele in the healthy (*p* < 0.05) and in the Alzheimer's disease groups of subjects (*p* < 0.1). As the levels of the BNP appear to be independent of the *APOE4* genotype in subjects with mild cognitive impairment, the results of our study support inclusion of measurements of plasma levels of the BNP in the list of the core Alzheimer's disease biomarkers for identification of the mild cognitive impairment group of patients. In addition, the results of our study warrant further investigations into molecular links between Alzheimer's disease-type cognitive decline and cardiovascular disorders.

## Introduction

Impairment in cognitive function or dementia is one of salient features of several age-related neurodegenerative disorders including Alzheimer's neurodegeneration. According to the most recent report published by the Alzheimer's Disease International organisation, it has been estimated that 46.8 million people worldwide were suffering from dementia in 2015 (World Alzheimer Report 2016: https://www.alz.co.uk/research/world-report-2016). Therefore, faced with large number of people suffering from decline in cognitive function worldwide, there is an urgent need for early and accurate detection of cognitive dysfunction. Protein biomarker(s) that could be measured from an easily accessible biological fluid, such as blood, would be an invaluable tool in serving as a) a diagnostic marker or b) a marker of disease progression, for an incurable neurodegenerative disorder such as Alzheimer's disease (AD) [1, 2].

Cardiovascular diseases (CVDs) as well as risk factors for CVDs (hypertension, dyslipidemia, diabetes mellitus) have been suggested to associate with poor cognitive function and may contribute to development of AD [3, 4, 5, 6, 7, 8, 9, 10]. Brain natriuretic peptide (BNP) and N-terminal pro-brain natriuretic peptide (NT-proBNP) are the most important humoral indicators of cardiac function and heart failure (HF) [10]. In addition, longitudinal monitoring of their values allows more accurate monitoring of HF progression as well as therapeutic efficacy of prescribed pharmacological therapy. Furthermore, based on the values of BNP and NT-proBNP, patients can be stratified into different clinical prognosis stage groups, regardless of their values being obtained for the left ventricular ejection fraction (LVEF) and other hemodynamic parameters (echocardiographic findings related to the left ventricle) [11].

Cardiomyocytes secrete BNP precursor which is synthesised as a prohormone, proBNP, containing 108 amino acids. ProBNP is proteolytically cleaved into biologically active hormone, the C-terminal part of the molecule, the BNP, containing 32 amino acids, and biologically inactive hormone, the N-terminal part of the molecule, the NT-proBNP, containing 76 amino acids. Although secreted and released in equimolar amounts, circulating plasma levels of NT-proBNP are higher than those of BNP and this is explained by the difference in their half-life, which is 20 minutes for BNP and 120 minutes for NT-proBNP [12, 13].

Apart from the fact that BNP has significantly shorter half-life than NT-proBNP, there is no significant difference between these natriuretic peptides and the optimal cut-off value for the diagnosis of HF is unclear for both BNP and NT-proBNP, although according to the guidelines of the European Society of Cardiology, patients presenting with BNP values that are < or = 35pg/ml and NT-proBNP values that are < or = 125pg/ml, in non-acute setting, and BNP values of < or = 100pg/ml and NT-proBNP values of < or = 300pg/ml, in acute setting, receive accurately ruled out diagnosis of heart failure [12, 14, 15, 16, 17]. However, overall, diagnostic utility of BNP fairs better than that of the NT-proBNP, as BNP values do not seem to be discordant between different commercially available assays being used, as do NT-proBNP values, especially for diagnoses of mild systolic left ventricular dysfunction (LVD) and isolated diastolic LVD [18].

Factors that appear to influence higher plasma levels of NT-proBNP are the following ones: advancing age, female gender, increased dyspnoea, diabetes mellitus, heart valve disease, heart rate reduction, LVEF value of < or = 45%, pathological electrocardiogram (ECG), high plasma creatinine concentration, low plasma glycosylated haemaglobin A1c (HbA1c) concentration and high urinary albumin concentration [19]. These factors have to be considered when evaluating NT-proBNP as well as BNP levels in diagnosing heart failure—it has been suggested that, for example, the cut off value for diagnosis of heart failure, in the elderly population, should be a BNP plasma concentration value of greater than > or = 400pg/ml [14, 15, 16, 20]. In addition, several drugs that are used for pharmacological treatment of HF (spironolactone, enalapril, valsartan) cause a decrease in BNP levels [21, 22, 23], although others (digoxin) appear to have the opposite effect [24].

In a targeted proteomic approach, several research groups have examined association between BNP and NT-proBNP and cognitive performance, in independently recruited and characterised cohorts of subjects [25, 26, 27, 28, 29, 30]. Some studies found that increased levels of BNP, measured in blood, were associated with reduced cognitive performance in subjects with no histories of neurologic or severe psychiatric disorders [25], and that increased BNP levels highly associated with the clinical diagnosis of very mild dementia, mild cognitive impairment (MCI) and AD, grouped together [28]. In other studies, BNP levels were found to be elevated in patients with subcortical vascular dementia but not in patients diagnosed with AD [26]. Furthermore, increased NT-proBNP levels were associated with poorer cognition in subjects with mental illness and in subjects who were diagnosed with MCI and AD [29, 30].

The most devastating characteristic of AD is a progressive loss of mental function. As previous studies examining plasma levels of BNP in association with AD-related cognitive decline reported results that are in disagreement with each other [26, 28], in this study, we investigated whether plasma measurements of BNP, that were obtained by using a novel, indirect approach for plasma peptide/protein detection called a SOMAscan [31], may predict a decline in cognitive function which occurs during AD progression. For our study, we utilised the so-called Open Science initiative whereby original data that was collected during the Europe-wide AddNeuroMed biomarker project is being shared with larger scientific community through the Accelerating Medicines Partnership—Alzheimer's Disease (AMP-AD) Knowledge Portal (https://www.nia.nih.gov/research/amp-ad).

## Materials and methods

### Subjects

Subjects were recruited for the European Union-funded AddNeuroMed biomarker project through seven medical centres based in different European countries (UK, Finland, Italy, Greece and Poland) [32, 33]. Informed consent was obtained for all subjects according to the Declaration of Helsinki (1991).

### Plasma samples

According to the AddNeuroMed's project protocol, all subjects were required to fast for a minimum of two hours prior to blood sample collection. Blood samples were collected into ethylenediaminetetraacetic acid (EDTA)-containing tubes. Following centrifugation, plasma supernatant was aliquoted into new tubes and stored at -80°C until further use [32, 33].

### BNP measurements

BNP levels in human plasma samples were measured using Slow Off-rate Aptamer (SOMAmer)-based capture array called SOMAscan, which was developed by SomaLogic, Inc., CO, USA. The SOMAscan uses a protein signal present in the human plasma and transforms it to a nucleotide signal that can be quantified using fluorescence on microarrays [31]. BNP levels, measured in human plasma samples by the SOMAscan, have been labelled as "BNP-32", denoting the 32 amino acid peptide sequence (SPKMVQGSGCFGRKMDRISSSSGLGCKVLRRH) deposited in the UniProt's database under a unique identifier PRO_0000001532 or P16860[103–134]. BNP measurements from the AddNeuroMed subjects were obtained through authorised access from the Synapse Data Platform, which is hosted by Sage Bionetworks (https://www.synapse.org/#!Synapse:syn2790911/wiki/235389), a part of the Accelerating Medicines Partnership—Alzheimer's Disease (AMP-AD) Knowledge Portal (https://www.nia.nih.gov/research/amp-ad), following ethical approval from the Sarajevo School of Science and Technology Institutional Review Board from the 5th of February 2018.

### Data cleaning and preprocessing

The obtained AddNeuroMed dataset contained 931 instances. This dataset can be accessed by its DOI number which is as follows—doi:10.7303/syn17021321, or it can be accessed from the Synapse Data Platform under the following URL - https://www.synapse.org/#!Synapse:syn17021321. Six variables that were selected for the purpose of this study's analyses were the following ones: diagnosis ("CTL" = control, healthy subjects, "MCI" = subjects diagnosed with mild cognitive impairment and "AD" = subjects diagnosed with probable Alzheimer’s disease), BNP-32 levels, age, polymorphism within the apolipoprotein E (*APOE*) gene, that is, absence or presence of the epsilon (ε) 4 allele or *APOE4* ("0" = absence or non-carrier, "1" = presence or carrier), sex/gender (female, male) and location/centre (seven nominal values).

Our examination focussed on BNP-32 levels that were measured from the first plasma samples taken from subjects at recruitment stage for a longitudinal study in seven different European centres, when their AD-related diagnosis was first established, according to a result of their Mini-Mental State Examination (MMSE) test, a 30-point questionnaire that is used for assessment of a subject's cognitive status [34]. Only subjects with a consistent diagnosis over the course of different evaluation time-points or visits were included in our study; that is, subjects whose diagnoses changed from, for example, "MCI" to "AD", during the course of the AddNeuroMed Project, were excluded from our analyses.

Outliers and missing data in our dataset were sufficiently present to possibly influence the change of data. Therefore, the raw dataset that has been obtained from the Synapse Data Platform (which can be accessed as described above) required cleaning and preprocessing, including data removal, missing data replacement and outlier cleaning.

**Fig 1:**
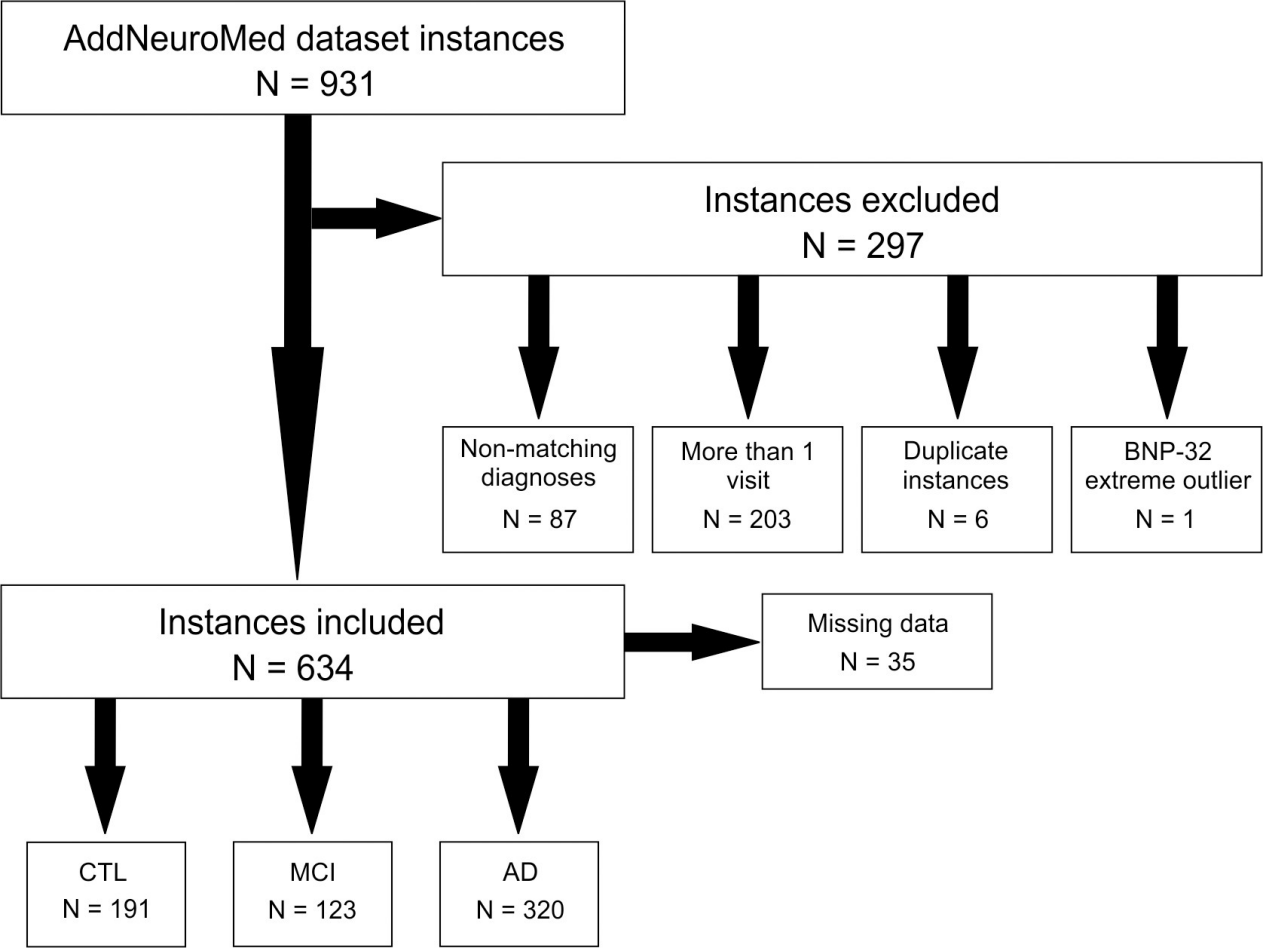
Details of subjects' inclusion or exclusion in the current analysis. Human subjects that were recruited and examined for the AddNeuroMed biomarker project were included or excluded in the current analysis based on the specific criteria, as described in Data cleaning and preprocessing sub-section of the Materials and methods section.

Given the size of the dataset, the aim was to preserve data instances, favouring data replacement to removal. Fig 1 shows the procedure that was followed in removing data instances, namely:

- 87 instances with non-matching diagnoses, at different evaluation time-points;
- 203 instances with a visit number other than the first visit;
- 6 duplicate instances;
- 1 extreme outlier, an exceedingly large BNP-32 value of 9452.96.

Outliers in BNP-32 levels (17 cases, grouped by diagnosis) were resolved by applying winsorization and trimming with a multiple imputation method. Winsorization was applied to 2 lower-range outliers (values: 2170.4539, 2362.8135), substituted with the closest lower bound value within the sample range, minus one unit (i.e., 2509 and 2508). The 15 upper-range outliers (values ≥ 3566.5000), of which 3 values were extreme outliers (values: 4051.0690, 4082.7000, 4137.8404), were first trimmed then replaced with imputed values (five iterations of imputation), using the regression method.

Missing values were present only in two variables—sex/gender (3 cases) and *APOE4* genotype (32 cases): these were also replaced using the multiple imputation method. Finally, the raw dataset displayed age of one of the subjects as a categorical value (89+), which was transformed to a numeric value of 90.

Dataset preparation and case removal were performed using Microsoft Excel software (Microsoft). IBM SPSS software was used for outlier and missing values cleaning (IBM Analytics).

### Statistical analysis

Data was analysed in IBM SPSS (IBM Analytics). Crosstab descriptive analysis was used to categorise subjects by age, sex/gender and *APOE4* presence into the three groups of diagnoses (Table 1).

**Table 1:** Demographic and clinical characteristics of study's subjects.

Subjects	Total (N = 634)		
	CTL (N = 191)	MCI (N = 123)	AD (N = 320)
**Age (mean ± SD)**	72.32 ± 5.26	76.71 ± 6.36	79.03 ± 6.69
**Sex (% female)**	52.9	60.7	69.2
***APOE4* (% carrier)**	25.9	30.6	56.6

According to their diagnoses, subjects were divided into three different groups: 1. "CTL" control or healthy subjects, 2. subjects that were diagnosed with mild cognitive impairment or "MCI" and 3. subjects that were diagnosed with probable Alzheimer's disease or "AD". Subjects' age is presented as an average or mean value with standard deviation or SD. Subjects' gender and subjects' inheritance of at least one ε 4 allele of the *APOE* gene (*APOE4*) are expressed as a percentage of the female gender and a percentage of the positive *APOE4* genotype, respectively.

Two types of inferential tests were run: mean comparison and regression. One-way ANOVA was used to compare BNP-32 levels between the three groups of diagnoses. In addition, an independent samples *t*-test was applied to examine a difference in BNP-32 between: 1) healthy, control subjects and 2) subjects with a cognitive decline (MCI subjects together with AD subjects); and between *APOE4* carriers and non-carriers, as well as female and male subjects, within each group of diagnosis. Finally, similar to the previously published study by Sattlecker and colleagues [32] who, for the first time, used the SOMAscan assay in a discovery study of plasma biomarkers of AD, multinomial logistic regression was used in exploring whether BNP-32 levels, age, *APOE4* genotype, sex/gender and geographical location of the medical centre can predict a decline in cognitive function.

## Results

In order to determine whether plasma measurements of BNP-32 reflect a decline in cognitive function associated with AD-type neurodegeneration, BNP-32 levels were analysed in subjects that were recruited and examined in seven different European medical centres, according to the criteria described in Data cleaning and preprocessing sub-section of the Materials and methods section. In total, BNP-32 values, as measured by the SOMAscan, were analysed in 634 subjects, grouped according to their diagnoses into 1. control or healthy subjects, 2. subjects who were diagnosed with MCI and 3. subjects who were diagnosed with probable AD (Table 1).

**Fig 2:**
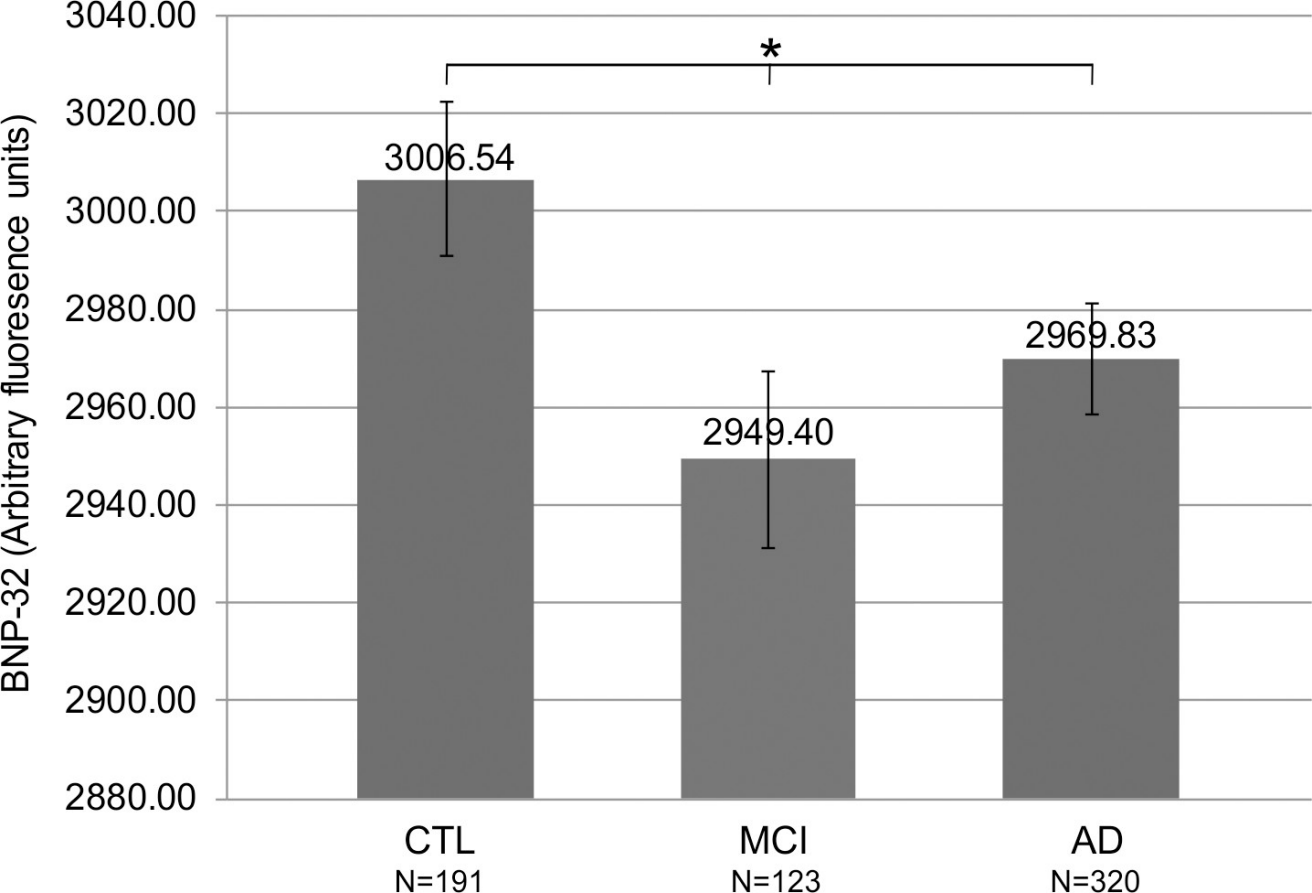
SOMAscan measured plasma BNP-32 levels are significantly different between healthy or control subjects, subjects with mild cognitive impairment and Alzheimer's disease patients. Average or mean BNP-32 levels, containing pooled SOMAscan data, after 5 imputation iterations (see Data cleaning and preprocessing section within Materials and methods) were compared between healthy, control subjects or CTL (3006.54±15.84), subjects with mild cognitive impairment or MCI (2949.40±17.95), and subjects with probable Alzheimer's disease or AD (2969.83±11.20). BNP-32 levels were significantly different between the three groups of subjects, as shown by a one-way ANOVA (*F*(2, 616) = 3.29, *p* < 0.05). (*: *p* < 0.05.).

According to a one-way ANOVA test, BNP-32 levels were significantly different between the three types of diagnoses (*F*(2, 616) = 3.29, *p* < 0.05), whereby MCI had the lowest mean BNP-32 value (2949.4, Std. Error = 17.96), and healthy subjects' BNP-32 was the highest (3006.54, Std. Error = 15.84) (Fig 2). BNP-32 levels were also significantly different (*t* = -2.4, *p* = 0.017) when comparing healthy subjects (3006.54, Std. Error = 15.84) to those diagnosed with MCI and AD, grouped together (2964.12, Std. Error = 9.48).

Multinomial logistic regression model was used in predicting a decline in cognitive function based on five independent factors: subject’s BNP-32 level, age, *APOE4* genotype, sex/gender and location of the recruiting/evaluation centre. The model had a satisfactory goodness-of-fit (*χ*^*2*^(1150) = 1079.03, *p* = 0.933), with the accuracy level of 63.6%. The considered factors significantly improve the model (*χ*^*2*^(20) = 231.67, *p* = 0.000) and explain 37.6% of variance in the diagnosis. The model indicates that all five factors can predict the diagnosis. BNP-32 level influences the difference between the healthy and the MCI diagnoses: as BNP-32 increases by a unit, the odds of being diagnosed with MCI decrease by 0.1% (*β* = -0.001, *p* = 0.019).

Age stimulates a decline in cognitive function, both in MCI and in AD. As age increases by a year, the odds of having AD (compared to being healthy) increase by 1.2 times (*β* = 0.182, *p* = 0.000), and the odds of having MCI increase by 1.07 times (*β* = 0.065, *p* = 0.002). Moreover, an increase in age decreases (by 11.1%) the odds of being diagnosed with MCI compared to AD (*β* = -0.117, *p* = 0.000).

**Fig 3:**
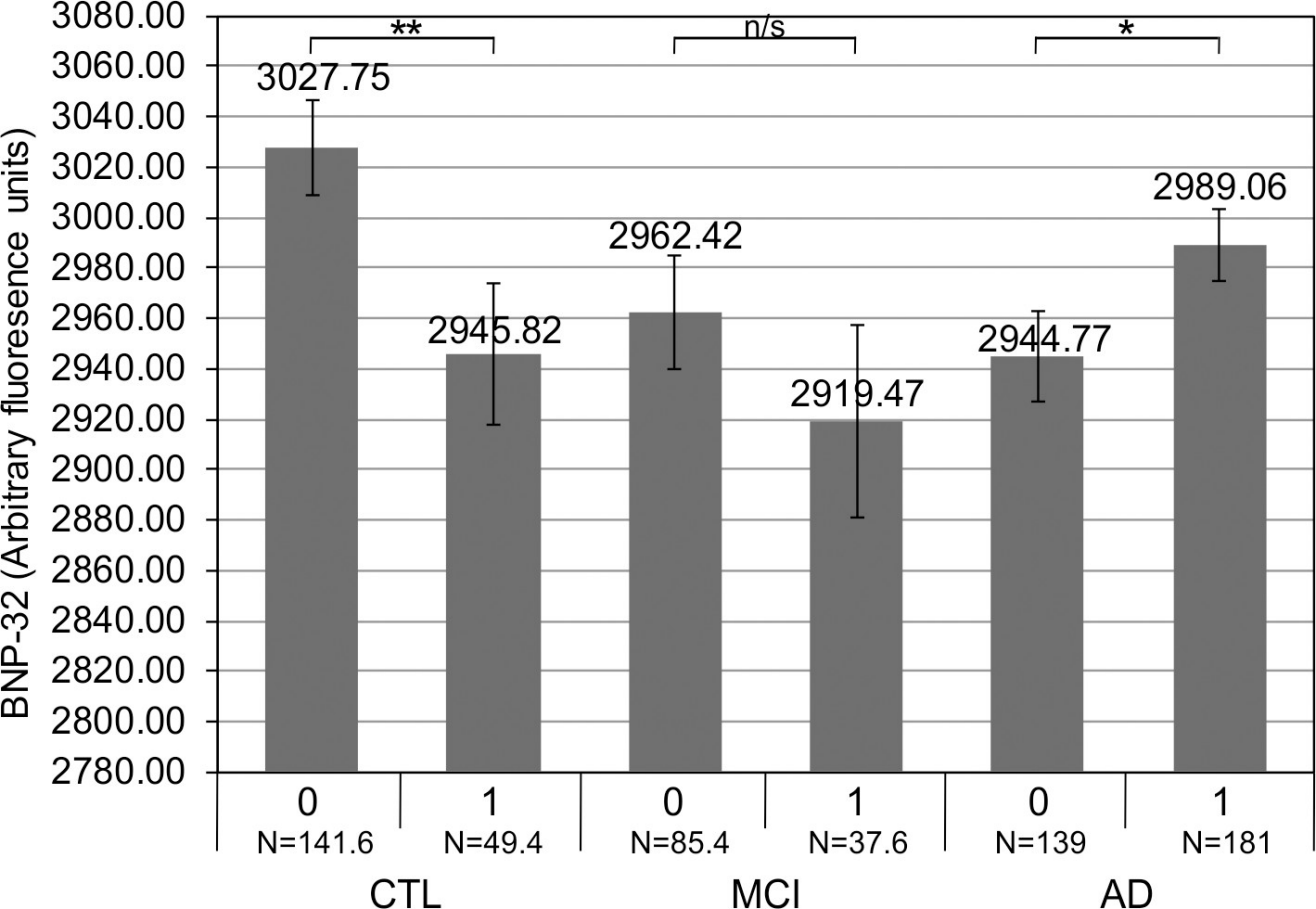
BNP-32 levels are significantly different between *APOE4* non-carriers and *APOE4* carriers in the healthy, control and the Alzheimer's disease groups of subjects. Average or mean BNP-32 levels, containing pooled SOMAscan data, after 5 imputation iterations (see Data cleaning and preprocessing section within Materials and methods), were compared within each group of subjects (1. healthy, control subjects or CTL, 2. subjects with mild cognitive impairment or MCI and 3. subjects with probable Alzheimer's disease or AD), between the *APOE4*-negative ("0") and the *APOE4*-positive ("1") subjects. Independent samples *t*-test was used to determine if there exists a statistically significant difference between different average values or means. A significant difference in BNP-32 levels when comparing *APOE4* non-carriers and *APOE4* carriers was established in healthy, control subjects and in subjects with probable AD. Differences were considered significant at a *p* value of < 0.05 and at < 0.1. Error bars represent a standard error. (**: *p* < 0.05; *: *p* < 0.1; n/s: non-significant.).

An *APOE4* non-carrier is less likely to be diagnosed with AD than as healthy (probability decreases by 79.6%, *β* = -1.588, *p* = 0.000), and is more likely to be diagnosed with MCI than with AD (odds increase 3.35 times, *β* = 1.21, *p* = 0.000). When examined in the entire dataset, which was divided according to the *APOE4* genotype, difference in BNP-32 levels—in *APOE4* carriers *vs APOE4* non-carriers—was not significant (*t* = 0.576, *p* = 0.565). However, when examined within each group of subjects categorised by diagnosis, while not significant within the group of subjects with MCI (*t* = 0.927, *p* = 0.359), the difference in BNP-32 levels between the *APOE4* non-carriers and the *APOE4* carriers was significant at *p* < 0.05 in the healthy, control subjects (*t*(7694) = 2.285, *p* = 0.022) and at *p* < 0.1 in the group of subjects with probable AD (*t*(1495) = -1.961, *p* = 0.050). The results, hence, imply that in healthy subjects the mean BNP-32 levels are significantly higher in *APOE4* non-carriers (3027.75, Std. Error = 18.96) than in *APOE4* carriers (2945.82, Std. Error = 27.68). It is furthermore likely (at *p* < 0.1) that in subjects with probable AD the opposite is true—mean BNP-32 levels are significantly lower in *APOE4* non-carriers (2944.77, Std. Error = 17.95) compared to *APOE4* carriers (2989.06, Std. Error = 14.08) (Fig 3).

**Fig 4:**
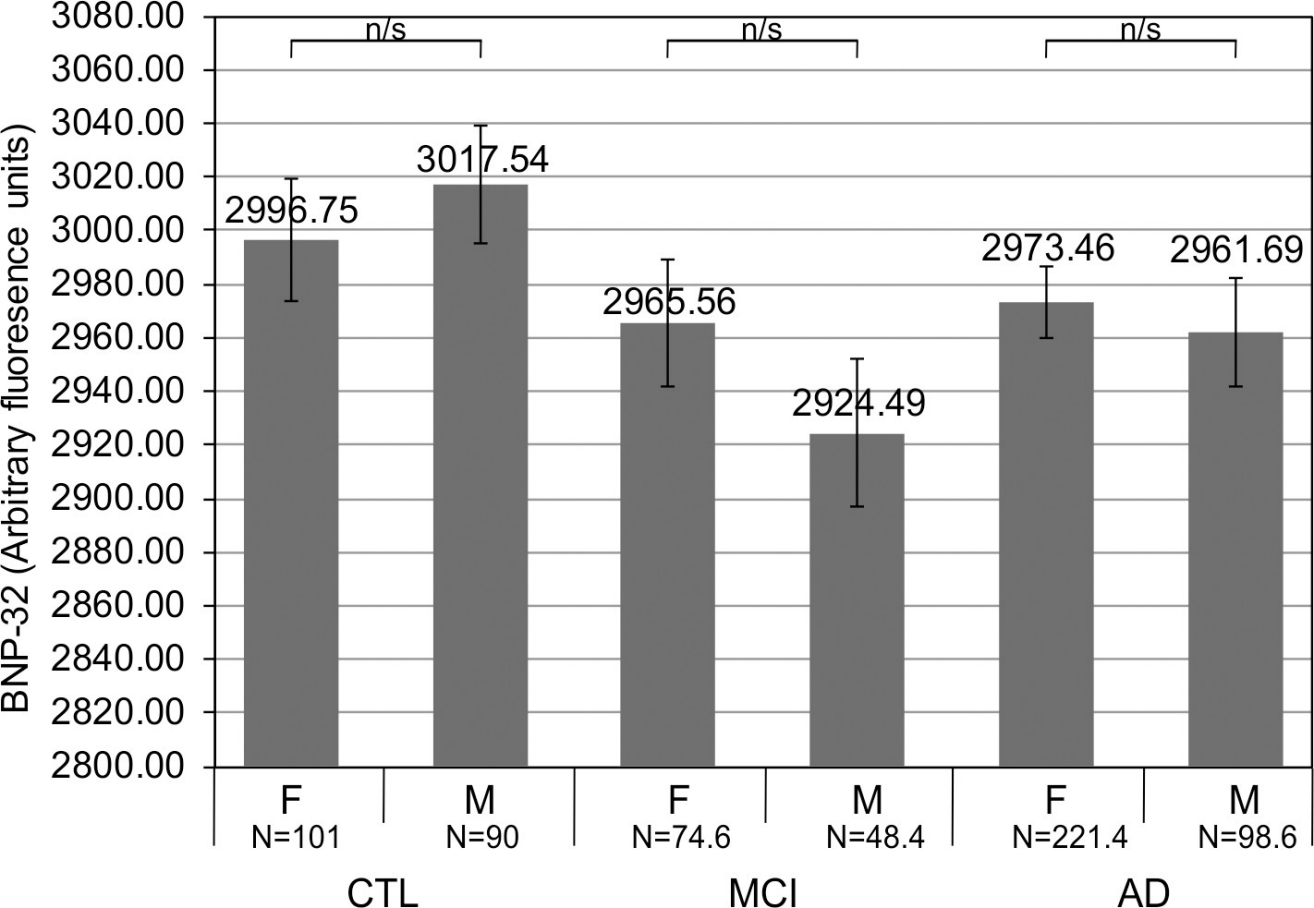
BNP-32 levels are not different between the female and the male subjects. Average or mean BNP-32 levels, containing pooled SOMAscan data, after 5 imputation iterations (see Data cleaning and preprocessing section within Materials and methods), were examined within each group of subjects (1. healthy, control subjects or CTL, 2. subjects with mild cognitive impairment or MCI and 3. subjects with probable Alzheimer's disease or AD), whereby the BNP-32 levels were compared between female ("F") and the male ("M") subjects. Independent samples *t*-test was used to determine if there exists a statistically significant difference between different average values or means. No statistically significant difference was present in BNP-32 levels between the female and the male subjects in any of the groups of subjects examined in this study. Error bars represent a standard error. (n/s: non-significant.).

The results of the multinomial logistic regression further indicated that females compared to males are 2.05 times more likely to be diagnosed with AD than as healthy (*β* = 0.719, *p* = 0.002). Females and males did not differ significantly in their BNP-32 levels (*t* = 0.154, *p* = 0.878), when the BNP-levels were analysed in the entire dataset, which was divided according to sex/gender. The results were also non-significant when examining the difference in BNP-32 levels between females and males within the three groups of diagnoses (1. healthy, control subjects (*t* = - 0.655, *p* = 0.513), 2. subjects with MCI (*t* = 1.119, *p* = 0.263) and 3. subjects with probable AD (*t* = 0.486, *p* = 0.627)) (Fig 4).

A further finding of the multinomial logistic regression was that a subject’s geographical location might significantly affect the odds of being diagnosed as healthy or with a probable AD—in favour of a healthy diagnosis.

## Discussion

Atherosclerosis, arteriosclerosis, hypertensive arteriopathy, endothelial dysfunction, impaired vascular autoregulation, heart failure, and anaemia belong to different types of cardiovascular pathologies that contribute to the development of dementia [35, 36, 37]. As we wished to investigate some of the molecular mechanisms involved in this process, we examined plasma levels of one of the established cardiac biomarkers, BNP, in a well-characterised cohort of subjects, with different dementia subtypes, that were recruited and examined in seven different medical centres in Europe, during the AddNeuroMed biomarker project and, therefore, represent a wide European population [32, 33]. Previous study performed by Kondziella and colleagues demonstrated that BNP levels were significantly increased in patients diagnosed with subcortical vascular dementia, but not in patients diagnosed with probable AD, when compared with age-matched healthy subjects [26]. In contrast, a study by Hu and colleagues examined BNP levels in the Alzheimer's Disease Neuroimaging Initiative (ADNI) cohort, which consisted of control or healthy subjects (N = 58), subjects diagnosed with MCI (N = 396) and subjects diagnosed with AD (N = 112), by using a multiplexed immunoassay (Rules-Based Medicine, Austin, TX, USA), and found elevated levels of BNP in patients with clinical MCI/AD compared to healthy, control subjects [28]. In our examination of BNP levels, we used the AddNeuroMed project dataset that has been deposited on the Synapse Data Platform in its raw form. Therefore, the data required cleaning and preprocessing, including data removal, missing data replacement and outlier cleaning (Fig 1). Outliers and missing values result from various factors including participant response errors and data entry errors [38]. Such data impurities are commonly dealt with three methods: trimming, replacing and robust estimation method [38]. Trimming represents a technique where outliers or missing data are removed by trimming a dataset. Winsorization involves replacing the outlier or missing values with expected values [38, 39]. Robust estimation method is used when the nature of the population distributions is known. As the latter does not apply to this dataset, methods that were applied were trimming and winsorization. It is often the case that clinical research (such as epidemiologic studies, multiple sclerosis, Parkinson’s disease, and AD studies, and reports having issues with incomplete datasets) increasingly employs various data preprocessing techniques, including winsorization, in resolving these issues [38, 39, 40, 41, 42, 43, 44, 45, 46], as it has been performed in the current study.

Therefore, in this study, a cohort of subjects for the BNP analysis consisted of healthy, control subjects (N = 191), subjects diagnosed with MCI (N = 123) and subjects diagnosed with probable AD (N = 320). Similar to the analysis performed by Hu and colleagues [28], who grouped subjects diagnosed with MCI and AD together (N = 508), and compared this group with healthy controls (N = 58), in our study, we also grouped together subjects with MCI and AD patients (N = 443), and compared their levels of BNP to the BNP levels detected in cognitively healthy individuals (N = 191). Our results are in contradiction to the results reported by Hu and colleagues: in our study, BNP levels were significantly reduced in dementia patients (MCI grouped with AD) compared with healthy subjects. When the BNP levels were compared between the three groups of subjects, across the AD spectrum, significant decrease in BNP, as measured by the SOMAscan assay, and calculated by a one-way ANOVA, was detected in the MCI subjects as well as in the AD patients, when compared with cognitively healthy individuals at a *p* < 0.05 (Fig 2). It may be possible that, in our study, significant decrease in BNP levels in the MCI and in the AD dementia subtypes are driven by the pharmacological antihypertensive therapy in these subjects, as previously demonstrated [21, 22, 23]. However, we were unable to establish whether the MCI and the AD subjects, who were recruited and examined for inclusion into the AddNeuroMed project, and evaluated in our study, were prescribed antihypertensive therapy at the time of their first evaluation, when their blood samples were first obtained and subsequently used in the SOMAscan assay.

As age, *APOE4* genotype and sex/gender are well-established risk factors for sporadic AD [47], we examined these parameters in this study. Firstly, it appears unlikely that our BNP findings are influenced by differences in the age between different groups of dementia subtypes, as previously shown [48], because our groups of subjects are well age-matched (Table 1). As for the status of the *APOE4* genotype, in our cohort, 56.6% of AD subjects have inherited at least one *APOE4* allele (Table 1), which is in close agreement with previously published findings that state that over 60% of persons with AD possess at least one *APOE4* allele [47]. Furthermore, the previously published prevalence of *APOE4* allele is 64% for MCIs and 51% for controls, respectively [49], which is different compared to the 30.6% for MCIs and 25.9% for controls in our cohort (Table 1). However, the presence of at least one of the alleles of the *APOE4* gene appears to influence BNP values in the healthy and the AD groups of subjects, whereas the BNP levels at the MCI stage of the disease appear to be independent of the *APOE4* genotype (Fig 3). Therefore, our results give rise to a hypothesis that *APOE4* genotype may exert a different kind of influence on the levels of the BNP at the extreme ends of AD progression, the cognitively healthy and the probable AD stage of the disease (Fig 3). Another possibility may be that, acting via an as yet unknown mechanism, the *APOE4* genotype may affect the activity of the BNP metabolising enzymes, including neprilysin and insulin degrading enzyme [50], which have also been demonstrated to regulate the metabolism of the amyloid β peptide, one of key molecules involved in the pathology of AD [51]. Moreover, as BNP is a potent vasodilator [52], one could speculate that physiological attempts at increasing BNP levels in the AD subjects who are *APOE4* carriers (Fig 3), may improve cerebral perfusion in a diseased brain, in case the peripheral BNP crosses the blood-brain-barrier thereby entering the brain to exert its vasodilatory effects.

As for sex/gender, in our cohort, 69.2% of females have a diagnosis of AD, which is in agreement with the well-documented sex/gender-based prevalence of AD which has been estimated to be over 60% in the female population, while the previously reported estimates of the female sex/gender prevalence in MCI and in healthy subjects (64% and 51%, respectively) are also similar to those present in our cohort (60.7% and 52.9%, respectively) (Table 1).

It is intriguing that the lowest BNP measurements have been recorded in the MCI subgroup. Mild cognitive impairment or MCI has been defined as the intermediate stage of a cognitive decline, between the changes seen in normal cognitive aging and those associated with advanced dementia/Alzheimer's disease, when a subject does not require help with the activities of daily living [53]. Patients with MCI represent a population of patients which is usually selected for pharmacological and non-pharmacological trials, testing novel treatments for AD, and some of the MCI patients exhibit longer-lasting or stable MCI. According to the AddNeuroMed data that were examined in this study, the MCI subjects remained within the MCI stage throughout a duration period of at least one year. Although, for diagnosing MCI subjects entering clinical trials, the core AD biomarkers (amyloid β_1–40_, amyloid β_1–42_, total tau and tau phosphorylated at serine 181 amino acid residue) are being measured—however, they can only be measured from a cerebrospinal fluid (CSF) sample, which is obtained from a patient by an invasive procedure—lumbar puncture. The results of our study, however, if confirmed by other research groups, support measuring BNP towards less-invasive identification of the MCI subgroup of patients by using blood. At present, we can only make assumptions about the molecular mechanism(s) that may lead to significantly decreased levels of plasma BNP in MCI subjects—based on an already published study that examined the activity of dipeptidyl peptidase-4 (DPP4) enzyme, one of several enzymes that degrade plasma BNP, as follows: it might be plausible that BNP levels in plasma samples of MCI subjects are significantly reduced, due to higher levels of DPP4 enzyme activity being present in the plasma samples of these subjects [54].

Overall, the molecular mechanism by which the BNP plays a role in AD-type cognitive impairment is unclear. This is partly because, based on the available evidence, it is not possible to speculate on the source of the physiological BNP being detected in human plasma (the heart *vs*. the brain). Kondziella and colleagues attempted to measure BNP in CSF and found it to be below the detection limit of the assay being used [26]. However, this may not exclude the BNP from having a role in the CNS. It may also suggest that the enzyme-linked immunosorbent assay (ELISA), that was used for its detection (SHIONORIA BNP kit, Shionogi, USA), has not been optimised for its utility of detecting BNP in CSF samples. In addition, it would be important to establish how the plasma BNP values, measured by the SOMAscan, correspond to the BNP concentrations measured by one of commercially available BNP ELISAs. This would enable establishing direct comparison between the SOMAscan BNP results with BNP values obtained in clinical practice for diagnosing specific stages of heart failure. Future studies should address these issues.
